# Cancer risk in patients with candidiasis: a nationwide population-based cohort study

**DOI:** 10.18632/oncotarget.18855

**Published:** 2017-06-29

**Authors:** Li-Min Chung, Ji-An Liang, Cheng-Li Lin, Li-Min Sun, Chia-Hung Kao

**Affiliations:** ^1^ Department of Medical Oncology, Zuoying Branch of Kaohsiung Armed Forces General Hospital, Kaohsiung, Taiwan; ^2^ Graduate Institute of Clinical Medical Science, School of Medicine, College of Medicine, China Medical University, Taichung, Taiwan; ^3^ Department of Radiation Oncology, China Medical University Hospital, Taichung, Taiwan; ^4^ Management Office for Health Data, China Medical University Hospital, Taichung, Taiwan; ^5^ College of Medicine, China Medical University, Taichung, Taiwan; ^6^ Department of Radiation Oncology, Zuoying Branch of Kaohsiung Armed Forces General Hospital, Kaohsiung, Taiwan; ^7^ Department of Nuclear Medicine and PET Center, China Medical University Hospital, Taichung, Taiwan; ^8^ Department of Bioinformatics and Medical Engineering, Asia University, Taichung, Taiwan

**Keywords:** candidiasis, cancer, population-based cohort study

## Abstract

**Background:**

Candidiasis and certain types of cancer are related to immunocompromised status. This study aimed to evaluate whether *Candida* infection (CI) is associated with subsequent cancer risk in Taiwan.

**Methods:**

Data from the National Health Insurance system of Taiwan were used to evaluate the association between CI and cancer risk. The CI cohort comprised 34,829 patients. Each patient was randomly frequency matched with one person from the general population without CI on the basis of age, sex, year of index date of CI diagnosis, and other characteristics to generate the control group. We used Cox's proportional hazard regression analysis to estimate the effects of CI on subsequent cancer risk.

**Results:**

Compared with the control group, patients with CI had a significantly higher risk of overall cancer (adjusted hazard ratio = 1.19, 95% confidence interval = 1.09–1.30). For subsite analysis, the risks of hematologic malignancy and head and neck, pancreatic, skin, and thyroid cancers were significantly higher in the CI group. Stratified analyses by sex, age, and follow-up time revealed different patterns.

**Conclusion:**

Our study suggested that CI can significantly increase overall and some individual cancer risks, which is partially compatible with previous findings.

## INTRODUCTION

Candidiasis, a fungal infection caused by yeasts of the genus *Candida*, is the most common oral fungal infection in humans. Among the at least 15 species of *Candida* yeasts that can infect humans, *Candida albicans* is the most prevalent [1, 2]. *Candida* species are commensal to healthy humans and are found frequently colonizing the oral mucosa. In addition, they are widespread elsewhere in the human body; however, *Candida* infection (CI) develops in immunocompromised situations due to the great adaptability of these species to different host niches [3]. CI frequently involves the mouth, vagina, glans penis, esophagus, liver, gastrointestinal tract, respiratory tract, and skin. Oral candidiasis is the most common type of fungal infection in the mouth [4] as well as the most common opportunistic oral infection in humans [5]. In Western countries, nearly 75% of adult women are affected by vaginal yeast infections at least once in their lives. Predisposing factors for CI include an impaired immune system and underlying disease states, drugs abuse and prolonged use of antibiotics, decreased digestive secretions, dietary factors, nutrient deficiency, impaired liver function, and altered bowel flora [6].

CI is not an infrequent complication of cancer and cancer-related therapy, and it may also play an active role in cancer development. The relationship between microbial infection and cancer is of great concern. Microbial infection and cancer risk have been investigated for decades, and some types of virus and bacteria are well-known carcinogens [7–11]. The consideration of candidiasis and certain types of cancer being related to the immunocompromised status of the host [12], scientists have also investigated the possible association between CI and subsequent cancer development, mostly focusing on oral cancers [13–15]. An earlier nationwide study in Denmark revealed that CI is associated with increases in both short- and long-term risks for several malignancies other than oral cancer [16]. The most recent findings demonstrate that *C. albicans* can promote cancer through several plausible mechanisms [17].

Using nationwide data, we conducted a similar population-based cohort study to evaluate whether CI increases overall and individual cancer risks in Taiwan. In addition, analyses stratified by sex, age, and follow-up time were conducted to determine whether significant findings are more likely to occur in certain groups.

## RESULTS

The characteristics of the CI and non-CI participants were similar and are listed in Table 1. The mean (SD) ages of patients in the CI and non-CI cohorts were 39.7 (15.4) and 41.3 (15.0) years, respectively.

**Table 1:** Baseline characteristics in individuals with and without candida infection

Characteristics	Candida infection		p-value
	No N = 34829 n (%)	Yes N = 34829 n (%)	
**Age group (year)**			0.99
20−49	27786(79.8)	27771(79.7)	
50−64	3979(11.4)	3988(11.5)	
≥ 65	3064(8.80)	3070(8.81)	
**Age, mean ±SD^a^ (year)**	41.3±15.0	39.7±15.4	0.001
**Sex**			0.85
Women	31721(91.1)	31707(91.0)	
Men	3108(8.92)	3122(8.96)	
**Monthly income^b^**			0.96
< 15,000	15697(45.1)	15703(45.1)	
15,000−19,999	10381(29.8)	10403(29.9)	
≥ 20,000	8751(25.1)	8718(25.0)	
**Urbanization level^c^**			0.99
1 (highest)	10349(29.7)	10328(29.7)	
2	10927(31.4)	10931(31.4)	
3	6068(17.4)	6077(17.5)	
4 (lowest)	7485(21.5)	7493(21.5)	
**Occupation^d^**			0.95
Office worker	18686(53.7)	18724(53.8)	
Laborer	13266(38.1)	13223(38.0)	
Other	2877(8.26)	2882(8.27)	
**Comorbidity**			
Chronic obstructive pulmonary disease	4158(11.9)	4188(12.0)	0.73
Connective tissue disease	450(1.29)	499(1.43)	0.11
Chronic renal failure	347(1.00)	380(1.09)	0.22
Chronic liver failure	3481(9.99)	3500(10.1)	0.81
Inflammatory bowel disease	391(1.12)	456(1.31)	0.02
Diabetes	1749(5.02)	1718(4.93)	0.59
Hypertension	5676(16.3)	5669(16.3)	0.94
Hyperlipidemia	4601(13.2)	4562(13.1)	0.66
HIV infection	88(0.25)	96(0.28)	0.55
Coronary artery disease	3178(9.12)	3196(9.18)	0.81
Drug dependence	41(0.12)	49(0.14)	0.40

SD, standard deviation;

Chi-square test;

^a^t-test

^b^Unit: New Taiwan dollar (NTD), 1 NTD is equal to 0.03US dollar;

^c^Urbanization level was categorized according to the population density of the residential area into 4 levels, with Level 1 the most urbanized and Level 4 the least urbanized.

^d^Other occupation category included primarily retired, unemployed, and low-income groups.

The incidence densities, adjusted hazard ratios (AHRs) between the CI and non-CI cohorts and 95% confidence intervals (CIns) are presented in Table 2. The overall incidence of all cancers was 17% higher in the CI cohort than in the non-CI cohort (3.93 vs. 3.73 per 1000 person-years) with an AHR of 1.19 (95% CIn = 1.09–1.30) in the following 14 years. Compared with the non-CI cohort, patients with CI had a significantly higher risk of hematologic malignancy (AHR=2.20, 95% CIn=1.52–3.18), myeloid leukemia (AHR=2.56, 95% CIn=1.28–5.12), non-Hodgkin's lymphoma (AHR=2.03, 95% CIn=1.22–3.39), head and neck cancer (AHR=2.43, 95% CIn=1.67–3.54), lip cancer (AHR=9.85, 95% CIn=1.25–77.9), oral cavity cancer (AHR=3.03, 95% CIn=1.82–5.04), pancreatic cancer (AHR=2.39, 95% CIn=1.09–5.24), skin cancer (AHR=2.35, 95% CIn=1.07–5.17), and thyroid cancer (AHR=1.45, 95% CIn=1.00–2.10).

**Table 2:** Incidences and hazard ratios of primary cancers between individuals with and without candida infection

Site of cancer	Candida infection				Crude HR (95 % CIn)	Adjusted HR (95 % CIn)
	No		Yes			
	Case	Rate^#^	Case	Rate^#^		
All cancers	919	3.73	1169	3.93	1.17(1.05, 1.30)**	1.19(1.09, 1.30)***
Hematologic malignancy	41	0.17	93	0.31	2.29(1.50, 3.49)***	2.20(1.52, 3.18)***
Hodgkin's disease	2	0.01	3	0.01	1.00(0.14, 7.10)	1.41(0.23, 8.48)
Lymphoblastic leukemia	2	0.01	7	0.02	3.50(0.73, 16.8)	3.57(0.74, 17.2)
Myeloid leukemia	11	0.04	30	0.10	2.88(1.29, 6.43)*	2.56(1.28, 5.12)**
Non-Hodgkin's lymphoma	22	0.09	46	0.15	2.27(1.24, 4.16)**	2.03(1.22, 3.39)**
Head and neck cancer	39	0.16	94	0.32	2.40(1.57, 3.67)***	2.43(1.67, 3.54)***
Lip	1	0.00	9	0.03	7.00(0/86, 56.9)	9.85(1.25, 77.9)*
Oral cavity	20	0.08	59	0.20	2.94(1.67, 5.18)***	3.03(1.82, 5.04)***
Oropharynx	5	0.02	5	0.02	3.00(0.31, 28.8)	0.97(0.28, 3.39)
Nasopharynx	12	0.05	16	0.05	1.00(0.43, 2.31)	1.23(0.58, 2.61)
Hypopharynx	1	0.00	3	0.01	2.00(0.18, 22.1)	3.29(0.34, 31.7)
Esophagus	6	0.02	12	0.04	1.33(0.46, 3.84)	2.14(0.80, 5.70)
Stomach	34	0.14	33	0.11	0.88(0.50, 1.56)	0.99(0.61, 1.61)
Colon, rectum	112	0.46	124	0.42	0.98(0.71, 1.33)	1.08(0.84, 1.40)
Liver	89	0.36	90	0.30	1.06(0.72, 1.58)	0.99(0.73, 1.33)
Pancreas	9	0.04	21	0.07	1.86(0.74, 4.65)	2.39(1.09, 5.24)*
Larynx	3	0.01	2	0.01	1.00(0.14, 7.10)	0.77(0.13, 4.67)
Lung	92	0.37	114	0.38	1.23(0.87, 1.74)	1.21(0.92, 1.60)
Malignant melanoma of skin	3	0.01	4	0.01	1.50(0.25, 8.98)	1.10(0.24,5.01)
Skin	9	0.04	21	0.07	1.43(0.54, 3.75)	2.35(1.07, 5.17)*
Breast cancer	225	0.91	260	0.87	0.95(0.76, 1.18)	0.98(0.81, 1.17)
Kaposi's sarcoma	0	0.00	2	0.01	-	-
Immune-related cancers	155	0.63	179	0.60	1.20(0.91, 1.58)	1.11(0.89, 1.38)
Cervix	41	0.17	37	0.12	0.80(0.47, 1.37)	0.81(0.52, 1.27)
Endometrium	31	0.13	27	0.09	0.73(0.38, 1.39)	0.72(0.43, 1.21)
Ovary	28	0.11	33	0.11	0.78(0.42, 1.45)	0.98(0.59, 1.62)
Prostate	27	0.11	15	0.05	1.00(0.43, 2.31)	0.70(0.37, 1.32)
Bladder, kidney	40	0.16	47	0.16	1.09(0.62, 1.92)	1.21(0.79, 1.85)
Brain	10	0.04	9	0.03	2.25(0.69, 7.31)	0.79(0.32, 1.95)
Thyroid	44	0.18	81	0.27	1.47(0.95, 2.27)	1.45(1.00, 2.10)*
Others	36	0.15	50	0.17	1.13(0.65, 1.98)	1.30(0.85, 2.01)

Rate^#^, incidence rate, per 1,000 person-years; Crude HR, relative hazard ratio; Adjusted HR^†^: multivariable analysis including age, sex, monthly-income, urbanization level, occupation, and comorbidity of chronic obstructive pulmonary disease, connective tissue disease, chronic renal failure, chronic liver failure, inflammatory bowel disease, diabetes, hypertension, hyperlipidemia, HIV infection, coronary artery disease, and drug dependence.

*p<0.05, **p<0.01, ***p<0.001.

Men with CI exhibited a significantly higher risk of all cancers, hematologic malignancy, head and neck cancer, and oral cavity cancer compared with men without CI (Table 3). Women with CI had 1.62- and 1.83-fold significantly higher risks of hematologic malignancy and non-Hodgkin's lymphoma, respectively, compared with women without CI.

**Table 3:** Cox model with hazard ratios and 95% confidence intervals of sub-division cancer associated with candida infection stratified by sex

Variable (ICD-9-CM)	Male with candida infection				Adjusted HR^†^ (95% CIn)	Female with candida infection				Adjusted HR^†^ (95% CIn)
	No (N=3108)		Yes (N=3122)			No (N=31721)		Yes (N=31707)		
	Case	Rate^#^	Case	Rate^#^		Case	Rate^#^	Case	Rate^#^	
All cancers	193	10.1	284	16.2	1.66(1.38, 2.00)***	726	3.20	885	3.16	1.07(0.97, 1.18)
Hematologic malignancy	5	0.26	30	1.71	6.35(2.46, 16.4)***	36	0.16	63	0.22	1.62(1.08, 2.45)*
Myeloid leukemia	0	0.00	13	0.74	-	11	0.05	17	0.06	1.30(0.61, 2.80)
Non-Hodgkin's lymphoma	4	0.21	11	0.63	2.98(0.95, 9.37)	18	0.08	35	0.12	1.83(1.03, 3.25)*
Head and neck cancer	16	0.83	70	3.99	4.73(2.75, 8.15)***	23	0.10	24	0.09	0.93(0.52, 1.65)
Lip	0	0.00	8	0.46	-	1	0.00	1	0.00	0.12(0.01, 20.1)
Oral cavity	10	0.52	52	2.96	5.60(2.84, 11.0)***	10	0.04	7	0.02	0.61(0.23, 1.61)
Pancreas	27	1.41	15	0.85	0.70(0.37, 1.32)	0	0.00	0	0.00	-
Skin	2	0.10	8	0.46	4.60(0.97, 21.7)	7	0.03	13	0.05	1.78(0.70, 4.51)
Thyroid	1	0.05	3	0.17	3.10(0.32, 30.0)	43	0.19	78	0.28	1.40(0.96, 2.04)

Rate^#^, incidence rate, per 1,000 person-years; Adjusted HR^†^: multivariable analysis including age, sex, monthly-income, urbanization level, occupation, and comorbidity of chronic obstructive pulmonary disease, connective tissue disease, chronic renal failure, chronic liver failure, inflammatory bowel disease, diabetes, hypertension, hyperlipidemia, HIV infection, coronary artery disease, and drug dependence.

*p<0.05, ***p<0.001.

Among the patients aged ≤49 years, those with CI were at a higher risk of hematologic malignancy and oral cavity cancer compared with those without CI (Table 4). Among the patients aged ≥50 years, those with CI were at a higher risk of all cancers, hematologic malignancy, myeloid leukemia, non-Hodgkin's lymphoma, head and neck cancer, lip cancer, oral cavity cancer, and thyroid cancer compared with those without CI.

**Table 4:** Cox model with hazard ratios and 95% confidence intervals of sub-division cancer associated with candida infection stratified by age

Variable (ICD-9-CM)	Age≤49 years		Age ≥50 years	
	Candida infection		Candida infection	
	No (N=27786)	Yes (N=27771)	No (N=7043)	Yes (N=7058)
	Adjusted HR^†^ (95%CIn)		Adjusted HR^†^ (95%CIn)	
All cancers	1(Reference)	0.96(0.85, 1.09)	1(Reference)	1.27(1.12, 1.43)***
Hematologic malignancy	1(Reference)	1.78(1.04, 3.04)*	1(Reference)	2.41(1.45, 4.02)***
Myeloid leukemia	1(Reference)	2.10(0.87, 5.08)	1(Reference)	3.24(1.05, 9.96)*
Non-Hodgkin'slymphoma	1(Reference)	1.61(0.72, 3.61)	1(Reference)	2.12(1.09, 4.09)*
Head and neck cancer	1(Reference)	1.58(0.93, 2.68)	1(Reference)	3.54(2.06, 6.10)***
Lip	1(Reference)	-	1(Reference)	9.65(1.22, 76.3)*
Oral cavity	1(Reference)	2.12(1.04, 4.34)*	1(Reference)	4.20(2.01, 8.75)***
Pancreas	1(Reference)	-	1(Reference)	0.72(0.38, 1.37)
Skin	1(Reference)	1.32(0.22, 8.13)	1(Reference)	2.35(0.97, 5.65)
Thyroid	1(Reference)	1.25(0.84, 1.87)	1(Reference)	2.93(1.07, 8.03)*

Adjusted HR^†^: multivariable analysis including age, sex, monthly-income, urbanization level, occupation, and comorbidity of chronic obstructive pulmonary disease, connective tissue disease, chronic renal failure, chronic liver failure, inflammatory bowel disease, diabetes, hypertension, hyperlipidemia, HIV infection, coronary artery disease, and drug dependence.

*p<0.05, ***p<0.001.

Furthermore, the AHRs for cancer types were stratified according to follow-up duration (Table 5). Among the patients with a follow-up duration of ≤1 year, those with CI exhibited a significantly higher risk of all cancers and non-Hodgkin's lymphoma compared with those without CI. Among the patients with 1 year < follow-up duration ≤5 years, those with CI had a 1.47-fold higher risk of all cancers, 2.64-fold higher risk of head and neck cancer, 2.35-fold higher risk of oral cavity cancer, and 4.05-fold significantly higher risk of thyroid cancer compared with those without CI. However, among the patients with a follow-up duration of >5 years, patients with CI had a 6.34-fold higher risk of skin cancer compared with those without CI.

**Table 5:** Cox model with hazard ratios and 95% confidence intervals of sub-division cancer associated with candida infection stratified by follow-up time

Variable(ICD-9-CM)	Follow-up time ≤1 year		1 year <Follow-up time ≤5 years		Follow-up time >5 years	
	Candida infection		Candida infection		Candida infection	
	No (N=481)	Yes (N=876)	No (N=11752)	Yes (N=6845)	No (N=22596)	Yes (N=27108)
	Adjusted HR^†^ (95%CIn)		Adjusted HR^†^ (95%CIn)		Adjusted HR^†^ (95%CIn)	
All cancers	1(Reference)	1.31(1.03, 1.67)*	1(Reference)	1.47(1.27, 1.69)***	1(Reference)	1.12(0.99, 1.28)
Hematologic malignancy	1(Reference)	7.15(0.97, 1.00)	1(Reference)	1.68(0.89, 3.15)	1(Reference)	1.34(073, 2.46)
Myeloid leukemia	1(Reference)	-	1(Reference)	2.17(0.69, 6.89)	1(Reference)	1.53(0.56, 4.20)
Non-Hodgkin's lymphoma	1(Reference)	6.39(1.84, 22.1)**	1(Reference)	1.69(0.66, 4.30)	1(Reference)	1.05(0.46, 2.38)
Head and neck cancer	1(Reference)	2.44(0.89, 6.69)	1(Reference)	2.64(1.54, 4.52)***	1(Reference)	1.16(0.56, 2.38)
Lip	1(Reference)	-	1(Reference)	4.93(0.54, 45.0)	1(Reference)	-
Oral cavity	1(Reference)	2.88(0.64, 13.0)	1(Reference)	2.35(1.15, 4.82)*	1(Reference)	1.68(0.61, 4.61)
Pancreas	1(Reference)	0.07(0.00, 1.51)	1(Reference)	1.13(0.45, 2.82)	1(Reference)	0.39(0.13, 1.19)
Skin	1(Reference)	0.34(0.02, 6.43)	1(Reference)	2.19(0.62, 7.78)	1(Reference)	6.34(1.43, 28.2)*
Thyroid	1(Reference)	0.82(0.29, 2.31)	1(Reference)	4.05(2.01, 8.18)***	1(Reference)	1.21(0.73, 2.00)

Adjusted HR^†^: multivariable analysis including age, sex, monthly-income, urbanization level, occupation, and comorbidity of chronic obstructive pulmonary disease, connective tissue disease, chronic renal failure, chronic liver failure, inflammatory bowel disease, diabetes, hypertension, hyperlipidemia, HIV infection, coronary artery disease, and drug dependence.

*p<0.05, **p<0.01, ***p<0.001.

Compared with the non-CI cohort, patients with candidiasis of the mouth exhibited a higher risk of all cancers (AHR = 2.24, 95% CIn = 1.91–2.63), hematologic malignancy(AHR = 9.54, 95% CIn = 5.79–15.7), myeloid leukemia (AHR = 14.0, 95% CIn = 5.71–34.3), non-Hodgkin's lymphoma (AHR = 8.92, 95% CIn = 4.49–17.7), head and neck cancer (AHR = 12.9, 95% CIn = 8.40–19.7), oral cavity cancer (AHR = 13.9, 95% CIn = 7.97–24.4), skin cancer (AHR = 4.99, 95% CIn = 1.72–14.5), and thyroid cancer (AHR = 2.75, 95% CIn = 1.22–6.20) (Table 6).

**Table 6:** Incidence rates and hazard ratios of primary cancers in subgroups of candida infection

Variables	N	Event	Rate^†^	Crude HR(95 % CIn)	Adjusted HR^†^(95 % CIn)
All cancers					
Control cohort	34829	919	3.73	1(Reference)	1(Reference)
Candidiasis of mouth	2156	195	13.5	3.61(3.10, 4.22)***	2.24(1.91, 2.63)***
Vulva and vagina	28072	669	2.61	0.67(0.61, 0.75)***	1.00(0.90, 1.11)
Other urogenital sites	544	24	9.20	2.51(1.67, 3.76)***	1.07(0.71, 1.61)
Hematologic malignancy					
Control cohort	34829	41	0.17	1(Reference)	1(Reference)
Candidiasis of mouth	2156	29	2.01	12.0(7.44, 19.3)***	9.54(5.79, 15.7)***
Vulva and vagina	28072	23	0.09	0.54(0.32, 0.90)*	0.68(0.40, 1.15)
Other urogenital sites	544	3	1.15	6.62(2.05, 21.4)**	3.41(1.03, 11.3)*
Myeloid leukemia					
Control cohort	34829	11	0.04	1(Reference)	1(Reference)
Candidiasis of mouth	2156	10	0.69	15.4(6.54, 36.3)***	14.0(5.71, 34.3)***
Vulva and vagina	28072	6	0.02	0.50(0.18, 1.35)	0.53(0.19, 1.45)
Other urogenital sites	544	0	0.00	-	-
Non-Hodgkin's lymphoma					
Control cohort	34829	22	0.09	1(Reference)	1(Reference)
Candidiasis of mouth	2156	15	1.04	11.5(5.97, 22.2)***	8.92(4.49, 17.7)***
Vulva and vagina	28072	14	0.05	0.61(0.31, 1.19)	0.89(0.44, 1.78)
Other urogenital sites	544	1	0.38	4.03(0.54, 29.9)	1.53(0.20, 11.6)
Head and neck cancer					
Control cohort	34829	39	0.16	1(Reference)	1(Reference)
Candidiasis of mouth	2156	66	4.57	28.6(19.3, 42.5)***	12.9(8.40, 19.7)***
Vulva and vagina	28072	12	0.05	0.32(0.17, 0.60)***	0.54(0.27,1.06)
Other urogenital sites	544	0	0.00	-	-
Lip cancer					
Control cohort	34829	1	0.00	1(Reference)	1(Reference)
Candidiasis of mouth	2156	8	0.55	-	-
Vulva and vagina	28072	0	0.00	-	-
Other urogenital sites	544	0	0.00	-	-
Oral cavity					
Control cohort	34829	20	0.08	1(Reference)	1(Reference)
Candidiasis of mouth	2156	45	3.11	37.9(22.4, 64.2)***	13.9(7.97, 24.4)***
Vulva and vagina	28072	4	0.02	0.21(0.07, 0.61)**	0.45(0.15, 1.40)
Other urogenital sites	544	0	0.00	-	-
Pancreas					
Control cohort	34829	9	0.04	1(Reference)	1(Reference)
Candidiasis of mouth	2156	1	0.07	1.89(0.24, 14.9)	1.04(0.13, 8.35)
Vulva and vagina	28072	12	0.05	1.25(0.52, 2.98)	2.51(0.95, 6.63)
Other urogenital sites	544	0	0.00	-	-
Skin					
Control cohort	34829	9	0.04	1(Reference)	1(Reference)
Candidiasis of mouth	2156	6	0.42	11.3(4.03, 31.8)***	4.99(1.72, 14.5)**
Vulva and vagina	28072	7	0.03	0.70(0.26, 1.90)	1.55(0.53, 4.57)
Other urogenital sites	544	1	0.38	10.7(1.35, 84.4)*	2.53(0.31, 20.7)
Thyroid					
Control cohort	34829	44	0.18	1(Reference)	1(Reference)
Candidiasis of mouth	2156	7	0.48	2.71(1.22, 6.03)*	2.75(1.22, 6.20)*
Vulva and vagina	28072	66	0.26	1.35(0.92, 1.99)	1.30(0.88, 1.92)
Other urogenital sites	544	0	0.00	-	-

Rate^#^, incidence rate, per 1,000 person-years; Crude HR, relative hazard ratio. Adjusted HR^†^, multivariable analysis including age, sex, monthly-income, urbanization level, occupation, and comorbidity of chronic obstructive pulmonary disease, connective tissue disease, chronic renal failure, chronic liver failure, inflammatory bowel disease, diabetes, hypertension, hyperlipidemia, HIV infection, coronary artery disease, and drug dependence.

*p<0.05, **p<0.01, ***p<0.001.

## DISCUSSION

The main finding of this study is that patients with CI had significantly higher risks of overall cancer as well as hematologic malignancy, head and neck cancer, pancreatic cancer, skin cancer, and thyroid cancer. According to stratified analyses, significantly higher risks were more likely observed in men, participants aged ≥50 years, and at a follow-up time between 1 and 5 years.

Several plausible mechanisms support the argument that *Candida* might induce cancer [17]. First, *Candida* can produce compounds such as nitrosamines, which are identified carcinogens that play a role in oral cancer initiation [18, 19]. Second, a previous study suggested that *C. albicans* promotes cancer through a proinflammatory response, mediated by an increase in cytokine production and adhesion-molecule expression [17]. It is increasingly clear that the tumor microenvironment, which is largely orchestrated by inflammatory cells, is an indispensable participant in the neoplastic process [20, 21]. Other hypotheses, such as the induction of Th17 response and molecular mimicry, have also been proposed to explain the mechanism by which *C. albicans* might promote cancer progression [17, 22, 23].

Nørgaard et al. used nationwide medical registries in Denmark to investigate the association between CI and cancer risk, observing an increase in both short- and long-term risks in all cancers; immune-related cancers; and cancers of several individual sites, such as the oral cavity, oropharynx, esophagus, larynx, and lung [16]. In our analyses, we adjusted for some chronic diseases that may be associated with immunosuppression. The results revealed significantly higher cancer risks among patients with CI for all cancers, hematologic malignancy, oral cavity cancer, lip cancer, pancreatic cancer, skin cancer, and thyroid cancer. Increased oral and lip cancer risks were expected on the basis of previous findings [13, 15–17]. CI and certain types of cancer are well-documented as being related to immunocompromised status [6, 12]. Immune-related cancers include liver, cervical, and anal cancers; malignant melanoma; non-Hodgkin's lymphoma; and Kaposi's sarcoma [12, 16]. Nørgaard et al. reported that patients with CI were at higher risks of all immune-related cancers pooled together and certain individual immune-related cancers, including non-Hodgkin's lymphoma and Kaposi's sarcoma [16]. By contrast, our analysis revealed that patients with CI had a significantly increased risk only for non-Hodgkin's lymphoma, and not for other immune-related cancers, either at individual sites or pooled together. Kaposi's sarcoma is relatively uncommon in Taiwan; we had only two cases in the CI cohort and no cases in the non-CI cohort. For other cancer sites, our results are partially consistent with the findings of Nørgaard et al., and revealed that patients with CI had an increased risk of oral cancer, lip cancer, and all head and neck cancers pooled together, in comparison with non-CI participants.

Recently, Zhu et al. reported that fungal infection promoted esophageal squamous cell carcinoma development in mice and that fungal infection was tightly associated with human esophageal squamous cell carcinoma [24]. Our data did not show a significant difference for the risk of esophageal cancer, and National Health Insurance Research Database (NHIRD) did not provide the pathological information for us to clarify if there is an increased risk of esophageal cancer if we separate squamous cell carcinoma from all the pathological types of esophageal cancer. By contrast, we unexpectedly detected significantly increased risks for pancreatic, skin, and thyroid cancers in patients with CI. We do not have sufficient evidence to explain this phenomenon; however, it may be partially related to the surveillance bias, particularly for skin and thyroid cancer. Patients with CI are assumed to visit dermatologists and head and neck specialists more frequently because of their infection sites (skin and oral cavity); consequently, the detection of more skin and thyroid cancers can be expected. The stratified analyses revealed different patterns in different categories. Although the majority of our patients with CI were women, male patients with CI were more likely to exhibit an increased risk of all cancers and individual cancers, particularly head and neck cancer and oral cavity cancer. Men tend to have poorer oral hygiene than women, which may increase the risk of both CI and head and neck cancers in men [25, 26]. Patients with CI aged 50 years or older are likely to exhibit more significant findings than younger patients with CI. Cancer is more common in elderly people, which may partially explain this finding. For the follow-up time, patients with CI with a follow-up time between 1 and 5 years exhibited a significantly higher risk of overall and individual cancers. However, a tendency of increased long-term risk of overall cancer was observed among patients with CI (95% CIn: 0.99–1.28).

The female: male ratio for our patients with CI was approximately 10:1, and the majority of our CI sites were the vulva and vagina. Therefore, we conducted a further analysis and specified candidiasis of the mouth, vulva and vagina, and other urogenital sites to assess whether cancer risk was specific to CI sites. Patients with candidiasis of the mouth exhibited a significantly increased risk of all cancers and individual cancers, except lip and pancreatic cancers (Table 6). By contrast, candidiasis of the vulva and vagina and other urogenital sites exhibited a considerably weaker association with overall or individual cancer risk.

The main strength of this study is the use of a nationwide database and longitudinal and comprehensive follow-up, which increased the generalizability of the findings. Furthermore, all National Health Insurance (NHI) claims are scrutinized by medical reimbursement specialists and peer reviewed to prevent errors and overutilization of medical resources; therefore, the diagnoses of CI and cancer are strongly validated. However, a few limitations must be acknowledged before our findings are interpreted. First, the NHI database lacks information on the life styles and health behaviors of patients, which prevented us from adjusting for potential confounding factors such as smoking and alcohol consumption in our analyses. Smoking and alcohol consumption are important predisposing factors for both CI [27, 28] and certain types of cancer [29]. Second, lack of individual microbiology data for CI and pathological data for tumor histology and staging in the NHIRD, so we could not identify the Candida species in patients with CI, which prevented us from conducting more sophisticated analyses to specify the role of C. albicans in the occurrence of cancer. We would not able to evaluate the relationship between CI infection and tumor histology/severity either. Third, possibly under-diagnosed or underestimated asymptomatic CI could exist, which might impact our results. Finally, surveillance bias, as mentioned earlier, could have distorted our results; however, this is less likely because more significant findings were detected during a follow-up time between 1 and 5 years, instead of less than 1 year (Table 5).

In conclusion, in this study, patients with CI exhibited higher risks of overall cancer as well as head and neck cancer, oral cancer, lip cancer, and non-Hodgkin's lymphoma, which is compatible with earlier reports. However, an unexpectedly high number of pancreatic, skin, and thyroid cancer cases were detected, and no confirmatory mechanisms could be identified to explain this phenomenon. Additional large-scale and comprehensive studies are warranted to support our findings.

## MATERIALS AND METHODS

### Data source

Approximately 99% of Taiwan's 23.74 million residents are covered by the NHI program, which was implemented in March 1995 [30]. The data in this retrospective cohort study were retrieved from the Taiwan Longitudinal Health Insurance Database2000 (LHID 2000). The data of one million people randomly selected from the medical claims records of the NHI 2000 Registry of Beneficiary enrollees were analyzed. The LHID2000 has been successfully used innumerous studies [31, 32].

### Ethics statement

The NHIRD encrypts patient personal information to protect privacy and provides researchers with anonymous identification numbers associated with relevant claims information, including sex, date of birth, medical services received, and prescriptions. Therefore, patient consent is not required to access the NHIRD. This study was approved to fulfill the condition for exemption by the Institutional Review Board (IRB) of China Medical University (CMUH104-REC2-115-CR1). The IRB also specifically waived the consent requirement.

### Sampled participants

**Figure 1:**
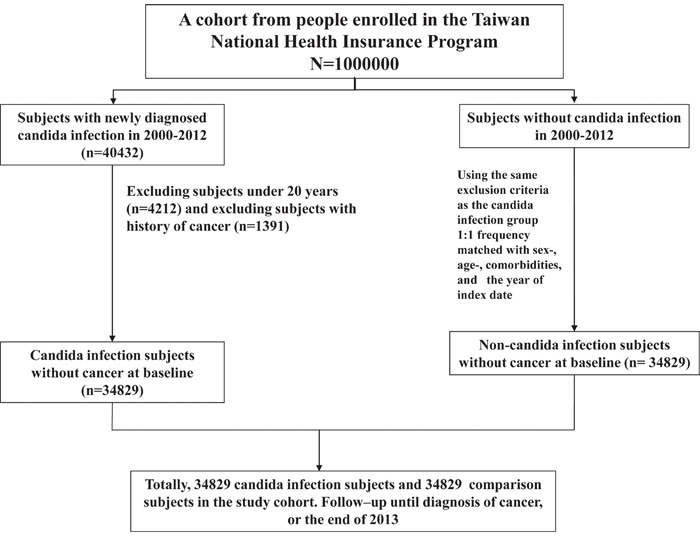
The selection process of the participants in the 2 study cohorts

Figure 1 shows the selection process of the participants in the 2 study cohorts. For the CI cohort, we selected patients aged ≥20 years and newly diagnosed as having CI [International Classification of Diseases (ICD)-9-CM code 112], including candidiasis of the mouth (ICD-9-CM code 112.0), vulva and vagina (ICD-9-CM code 112.1), other urogenital sites, and other sites, from January 1, 2000 to December 31, 2012. The CI diagnosis date was defined as the index date. Participants without CI were selected from the LHID2000 as the non-CI cohort. Patients in both the CI and non-CI cohorts who had previously received a diagnosis of cancer (ICD-9-CM code 140–208) were excluded. For each patient with CI, one non-CI control was frequency matched by age, sex, monthly income, urbanization level, occupation, year of index date, and comorbidity (chronic obstructive pulmonary disease, connective tissue disease, chronic renal failure, chronic liver failure, inflammatory bowel disease, diabetes, hypertension, hyperlipidemia, HIV infection, coronary artery disease, and drug dependence).

### Outcome measurement

We obtained data on patients who were diagnosed with cancer (ICD-9-CM codes 140–195, and 200–208) during 2000–2013 from the Registry for Catastrophic Illness Patient Database (RCIPD). Cancer registration in the RCIPD requires histological or pathological confirmation or a physician's confirmation. All participants were followed up from the index date until the date of cancer diagnosis, death, withdrawal from the NHI program, or the end of 2013.

### Statistical analysis

We first compared the distribution differences of sociodemographic variables and baseline comorbidities between the CI and non-CI cohorts by using the chi-square test for categorical variables and *t* test for continuous variables. The follow-up person-years were calculated to assess incidence density rates until the cancer was either identified or censored. Univariable and multivariable Cox proportional hazards models were used to estimate the hazard ratios and 95% CIns of the risk of cancer associated with CI compared with the non-CI cohort. The multivariable models were simultaneously adjusted for age, sex, monthly income, urbanization level, occupation, and comorbidities. Models were also used for estimating the risks of cancer stratified by sex, age group, and follow-up period. Further analysis was performed to assess whether the type of CI had a role in cancer outcomes. All analyses were performed using SAS for Windows (Version 9.4; SAS Institute, Inc., Cary, NC, USA), and the significance level was set at 0.05.
